# Imported Malaria in Children in Industrialized Countries, 1992–2002

**DOI:** 10.3201/eid1502.080712

**Published:** 2009-02

**Authors:** Katrin Stäger, Fabrice Legros, Gérard Krause, Nicola Low, David Bradley, Meghna Desai, Simone Graf, Stefania D’Amato, Yasutaka Mizuno, Ragnhild Janzon, Eskild Petersen, John Kester, Robert Steffen, Patricia Schlagenhauf

**Affiliations:** University of Zurich, Zurich, Switzerland (K. Stäger, R. Steffen, P. Schlagenhauf); Centre National de Référence de l'Epidémiologie du Paludisme d'Importation et Autochthone, Paris, France (F. Legros); Robert Koch Institut, Berlin, Germany (G. Krause); University of Bern, Bern, Switzerland (N. Low); Health Protection Agency, London, UK (D. Bradley); Centers for Disease Control and Prevention, Atlanta, Georgia, USA (M. Desai); Swiss Federal Office of Public Health, Bern (S. Graf); Ministero della Salute, Rome, Italy (S. D’Amato); Jikei University School of Medicine, Tokyo, Japan (Y. Mizuno); Swedish Institute for Infectious Disease Control, Solna, Sweden (R. Janzon); Statens Serum Institut, Copenhagen, Denmark (E. Petersen); United Nations World Tourism Organization, Madrid, Spain (J. Kester)

**Keywords:** pediatric, malaria, children, travel, research

## Abstract

Children account for an appreciable proportion of total imported malaria cases, yet few studies have quantified these cases, identified trends, or suggested evidence-based prevention strategies for this group of travelers. We therefore sought to identify numbers of cases and deaths, *Plasmodium* species, place of malaria acquisition, preventive measures used, and national origin of malaria in children. We analyzed retrospective data from Australia, Denmark, France, Germany, Italy, Japan, the Netherlands, Sweden, Switzerland, the United Kingdom, and the United States and data provided by the United Nations World Tourism Organization. During 1992–2002, >17,000 cases of imported malaria in children were reported in 11 countries where malaria is not endemic; most (>70%) had been acquired in Africa. Returning to country of origin to visit friends and relatives was a risk factor. Malaria prevention for children should be a responsibility of healthcare providers and should be subsidized for low-income travelers to high-risk areas.

## Abstract 2

Children account for a considerable proportion of cases imported to the United States and Europe.

Malaria is associated with high healthcare costs (*1*). Although several industrialized countries (e.g., most European countries, the United States, Australia, Japan) (*2*) are classified as malaria nonendemic, human migration and tourist travel to malaria-endemic regions are resulting in the importation of malaria (an estimated 30,000 cases/year) into these malaria-free countries (*3*). In the World Health Organization European Region, the number of imported cases rose from 1,500 in 1972 to 13,000 in 1999 (*4*). Children account for a considerable proportion of total malaria cases imported into the United States and Europe (*5*). International migration increased from 75 million in 1960 to 175 million in 2000 (*6*). Furthermore, the World Population Prospects report predicts a sharp increase in the number of persons who will migrate from southern (malaria-endemic) areas to northern (malaria-free) industrialized areas worldwide (*7*). This immigration trend also predetermines subsequent immigrant travel patterns. Immigrants returning to visit families in their home countries are at high risk for travel-related illness (*8*–*12*).

Malaria cases in children especially are increasing as more children travel and as the profile of immigrants changes (*13*–*16*). Our main study objectives were to evaluate the epidemiology of imported malaria in children in industrialized countries, identify trends and risk groups, and rank destinations according to malaria risk for children.

## Methods

We collected retrospective data on imported malaria cases in children for the 11-year period January 1992 through December 2002. We requested data from 11 industrialized countries in which malaria is not endemic: Australia, Denmark, France, Germany, Italy, Japan, the Netherlands, Sweden, Switzerland, the United Kingdom, and the United States.

### Malaria Cases

We defined imported malaria in a child as parasitologically confirmed malaria that had been acquired in a disease-endemic area by a person <18 years of age and that was diagnosed after clinical disease had developed and when the person was in an industrialized country where the disease was not endemic. Data were collated directly from the countries’ health authorities and consisted of aggregated case numbers of imported malaria in children by year (1992–2002), age group, sex, *Plasmodium* species, place of infection acquisition, number of deaths, national origin of patients, and preventive measures used during travel (i.e., chemoprophylaxis, bed nets, protective clothing, repellents).

We examined the distribution of malaria cases in children according to the variables of interest. Total cases were stratified according to *Plasmodium* species. On the basis of numbers of deaths and of *Plasmodium falciparum* cases, we calculated case-fatality ratio and 95% confidence intervals (CIs).

### Traveler Statistics

The United Nations World Tourism Organization provided data on total numbers of travelers and extrapolated numbers of children <18 years of age who had traveled from >1 of the 11 industrialized countries in this study to malaria-endemic areas in Africa. Because exact numbers were not readily available, we assumed and used as proxy data the proportion of young travelers from the overall number of arrivals at specific African destinations. The assumption that 1 of 10 travelers is <18 years of age is consistent with the number of visits of young UK residents to African destinations in 2000. During that year, an estimated 138,000 persons in this age group accounted for 1,439,000 visits to Africa (*17*). The number of malaria cases in children per 10,000 visitors can be treated as a proxy. Using the number of child travelers as denominator, we calculated the rate of malaria cases acquired by children in the African regions and compared destination countries in Africa according to malaria risk for young travelers. Denominator data did not account for time spent in the malaria-transmission area. We also used surveillance data to attempt to determine nationality or country of origin (ethnicity) of the children with malaria.

## Results

### Number of Cases

Of 17,009 reported malaria cases in children from the 11 industrialized countries studied, >75% were from only 3 countries (Table 1): France (n = 6,618), United Kingdom (n = 3,816; children <17 years of age), and United States (n = 2,614). The number of reported cases per year varied from 0 in Japan in 1996 to 1,096 in France in 1999. The number of cases registered in all contributing countries together was highest in 1999 (n = 2,233) and declined thereafter.

**Table 1:** Number of imported malaria cases in children in 11 industrialized countries, by year, 1992–2002*

Country	Year											Mean no. cases/y	Total no. cases
	1992	1993	1994	1995	1996	1997	1998	1999	2000	2001	2002		
Australia	162	144	138	111	183	160	147	158	119	107	75	137	1,504
Denmark	18	19	26	26	21	18	14	27	35	29	24	23	257
France	102	124	157	255	519	617	738	1,096	1,078	979	953	602	6,618
Germany	NA	41	75	65	96	77	57	93	68	113	72	76	757
Italy	NA	NA	NA	NA	NA	NA	93	72	69	96	77	81	407
Japan	5	4	5	9	0	4	1	6	5	2	4	4	45
Netherlands	13	22	17	43	41	36	37	35	79	57	40	38	420
Sweden	NA	NA	NA	NA	NA	31	21	31	16	20	36	26	155
Switzerland	37	51	37	34	41	49	32	35	34	36	30	38	416
United Kingdom	284	321	296	352	469	358	353	363	333	350	337	347	3,816
United States	159	206	173	215	293	333	253	317	227	263	175	238	2,614
Total cases	780	932	924	1,110	1,663	1,683	1,746	2,233	2,063	2,052	1,823		17,009

*All children were <18 years of age, except in the United Kingdom, where data were available only for children <17 years of age. NA, data not available.

Among the different age groups, the largest overall percentage of cases occurred in those 15–17 years of age (18.1% of total cases) (Table 2). Analysis by age group showed heterogeneity between the countries. Japan and Australia showed high case rates; the 18-year age group accounted for almost 25% and 15% of all cases, respectively. Boys accounted for 55% of the total cases and predominated in all participating countries (data not shown).

**Table 2:** Number of imported malaria cases in children in 11 industrialized countries, by age group, 1993–2002

Country	Age group, y, no. (%)*							Total
	0–2	3–5	6–8	9–11	12–14	15–17	18	
Australia	131 (8.7)	143 (9.5)	139 (9.2)	174 (11.6)	233 (15.5)	456 (30.3)	228 (15.2)	1,504
Denmark†	26 (10.1)	44 (17.1)	47 (18.3)	37 (14.4)	30 (11.7)	55 (21.4)	15 (5.8)	257
France	1,149 (17.4)	1,247 (18.8)	1,054 (15.9)	1,042 (15.7)	920 (13.9)	887 (13.4)	319 (4.8)	6,618
Germany‡	112 (14.8)	123 (16.2)	120 (15.9)	117 (15.5)	97 (12.8)	142 (18.8)	46 (6.1)	757
Italy§	80 (19.7)	107 (26.3)	67 (16.5)	47 (11.5)	31 (7.6)	57 (14.0)	18 (4.4)	407
Japan	1 (2.2)	8 (17.8)	10 (22.2)	1 (2.2)	6 (13.3)	8 (17.8)	11 (24.4)	45
Netherlands	47 (11.2)	67 (16.0)	65 (15.5)	45 (10.7)	45 (10.7)	115 (27.4)	36 (8.6)	420
Sweden¶	12 (7.7)	20 (12.9)	26 (16.8)	30 (19.4)	24 (15.5)	32 (20.6)	11 (7.1)	155
Switzerland	60 (14.4)	81 (19.5)	72 (17.3)	65 (15.6)	55 (13.2)	60 (14.4)	23 (5.5)	416
United Kingdom	351 (9.2)	619 (16.2)	621 (16.3)	714 (18.7)	707 (18.5)	804 (21.1)	Not available	3,816
United States	333 (12.7)	463 (17.7)	409 (15.6)	386 (14.8)	399 (15.3)	463 (17.7)	161 (6.2)	2,614
Total cases	2,302 (13.5)	2,922 (17.2)	2,630 (15.5)	2,658 (15.6)	2,547 (15.0)	3,079 (18.1)	868 (5.1)	17,009

*Percentage of total cases in children <18 years of age (<17 years of age for United Kingdom). †Data include 3 (1.2%) cases for which age was not specified. ‡Data from 1993–2002 only. §Data from 1998–2002 only. ¶Data from 1997–2002 only.

### Region of Malaria Acquisition

Of the 15,505 cases for which detailed data on country of acquisition were obtained, for all countries except Japan, >50% of cases were imported from Africa (Table 3); West Africa accounted for >50% of cases imported from Africa. Asia and Central and South America accounted for a small proportion of the imported malaria cases in children. Central and South America were responsible for a negligible number of infections, but in the United States, children accounted for 348 imported malaria cases (13% of all malaria cases in children [data not shown]). The predominant source of infections acquired in Asia was southern Asia (e.g., India, Pakistan, Sri Lanka) rather than Southeast Asia (e.g., Thailand, Indonesia, Vietnam, Malaysia, Philippines) (data not shown).

**Table 3:** Case rates for children in industrialized countries with malaria imported from Africa, 1992–2002*

Country of origin†	Region of case acquisition													
	Western Africa			Eastern Africa			Central Africa			Southern Africa			All African regions	
	No.	Rate (95% CI)		No.	Rate (95% CI)		No.	Rate (95% CI)		No.	Rate (95% CI)		No.	Rate (95% CI)
Denmark	46	47.7 (34.9–63.6)		87	54.7 (43.9–67.5)		10	434.8 (208.5–799.6)		1	0.8 (0.02–4.5)		144	37.8 (31.9–44.5)
France	3,777	110.6 (107.1–114.2)		1,339	25.7 (24.4–27.1)		1,400	216.3 (205.1–228.0)		1	0.1 (0.001–0.7)		6,517	65.0 (63.4–66.6)
Germany‡	344	38.3 (34.3–42.5)		129	4.6 (3.8–5.4)		76	68.0 (53.6–85.2)		5	0.2 (0.07–0.5)		554	8.9 (8.2–9.7)
Italy§	323	40.2 (35.9–44.8)		24	2.0 (1.3–2.9)		31	39.5 (26.8–56.1)		1	0.3 (0.006–1.4)		379	15.2 (13.7–16.8)
Japan	8	7.0 (3.0–13.8)		3	1.1 (0.2–3.1)		4	102.6 (27.9–262.6)		1	0.5 (0.01–2.6)		16	2.6 (1.5–4.2)
Netherlands	172	35.3 (30.2–41.0)		32	0.6 (0.4 – 0.9)		51	103.9 (77.3–136.6)		1	0.1 (0.0–0.7)		256	13.9 (12.2–15.7)
Sweden¶	55	51.1 (38.5–66.5)		48	9.0 (6.6–11.9)		21	244.2 (151.2–373.3)		0	0.0 (0.0–1.7)		124	14.9 (12.4–17.8)
Switzerland	97	54.9 (44.5–67.0)		37	4.4 (3.1–6.1)		85	121.6 (97.2–150.4)		2	0.5 (0.06–1.9)		221	15.0 (13.1–17.1)
United Kingdom	1,749	177.3 (169.0–185.8)		406	9.7 (8.8–10.7)		91	82.3 (66.8–101.1)		14	0.4 (0.2–0.7)		2,260	26.3 (25.2–27.4)
United States	1,181	160.6 (151.6–170.1)		191	9.9 (8.5–11.4)		91	62.6 (50.4–76.8)		8	0.5 (0.2–1.0)		1471	33.7 (32.0–35.5)
Total	7,752	99.1 (96.9–101.3)		2,296	13.0 (12.5–13.5)		1,860	151.6 (144.8–158.6)		34	0.3 (0.2–0.5)		11,942	32.4 (31.8–33.0)

*Rates per 10,000 arrivals of children <18 years of age, except in the United Kingdom, where data were available only for children <17 years of age. Regions are classified according to United Nations World Tourist Organization (*18*). Denominators include only countries for which data were available. No data were available for arrivals to Gabon and Burundi for any country. For some African countries data are available for only some sources, e.g., arrivals to Sierra Leone available for only Denmark, United Kingdom; arrivals to Mozambique available for only United Kingdom, United States (details available on request). Western Africa comprises Benin, Burkina Faso, Cape Verde, Ghana, Guinea, Guinea Bissau, Côte d’Ivoire, Liberia, Mali, Mauritania, Niger, Nigeria, Senegal, Sierra Leone, The Gambia, and Togo; Eastern Africa comprises Burundi, Comoros, Djibouti, Eritrea, Ethiopia, Kenya, Madagascar, Malawi, Mauritius, Mozambique, Reunion, Rwanda, Seychelles, Somalia, Sudan, Tanzania, Uganda, Zambia, and Zimbabwe; Central Africa comprises Angola, Cameroon, Central African Republic, Chad, Congo, Democratic Republic of the Congo (Zaire), Equatorial Guinea, Gabon; Sao Tome et Principe; and Southern Africa comprises Botswana, Lesotho, Namibia, South Africa, and Swaziland. †No data available from Australia. ‡Data from 1993–2002 only. §Data from 1998–2002 only. ¶Data from 1997–2002 only.

### Case-Fatality Ratio

The case-fatality ratio for all countries was <0.4%; 3 countries (Italy, Sweden, and Japan) recorded no malaria-associated deaths in children during the period of observation (Table 4). Information about use of chemoprophylaxis in traveling children was limited. Among children with malaria, only 17.5% had taken chemoprophylaxis.

**Table 4:** Case-fatality ratios for children with imported malaria in 8 of 11 industrialized countries, 1992–2002*

Country	Total no. cases	No. deaths	Ratio (95% confidence interval)
France	4,893	10	0.20 (0.09–0.37)
Germany†	512	2	0.39 (0.05–1.40)
Italy‡	335	0	0.00 (0.00–0.89)
Japan	15	0	0.00 (0.00–18.10)
Sweden§	93	0	0.00 (0.00–3.17)
Switzerland	273	1	0.37 (0.01–2.02)
United Kingdom	2,502	5	0.20 (0.06–0.47)
United States	1,225	4	0.33 (0.09–0.83)

*All cases caused by *Plasmodium falciparum.* Children were <18 years of age, except in France and the United Kingdom, where data were available only for children <15 and <17 years of age, respectively. No data were available from Australia, Denmark, and the Netherlands. †Data from 1993–2002 only. ‡Data from 1998–2002 only. §Data from 1997–2002 only.

### *Plasmodium* Species

*Plasmodium* species varied among the countries. The predominant species was *P. falciparum*, which accounted for 69.9% of all cases. The highest proportion of cases caused by *P. falciparum* (83.1%) was in France (Table 5).

**Table 5:** *Plasmodium* species causing imported malaria in children in 10 of 11 industrialized countries, 1992–2002*

Country	Total no. cases						
	Including cases caused by mixed or unknown species	Excluding cases caused by mixed or unknown species		Cases caused by known species, no. (%)			
				*P. falciparum*	*P. vivax*	*P. ovale*	*P. malariae*
Denmark	257	243		146 (56.8)	74 (28.8)	17 (6.6)	6 (2.3)
France	6,618	6,275		5,502 (83.1)	282 (4.3)	365 (5.5)	126 (1.9)
Germany†	757	685		512 (67.7)	140 (18.5)	16 (2.1)	17 (2.2)
Italy‡	407	402		335 (82.3)	34 (8.4)	19 (4.7)	14 (3.4)
Japan	45	40		15 (33.3)	19 (42.2)	5 (11.1)	1 (2.2)
Netherlands	420	349		237 (56.4)	74 (17.6)	23 (5.5)	15 (3.6)
Sweden§	155	142		93 (60.0)	33 (21.3)	9 (5.8)	7 (4.5)
Switzerland	416	368		273 (65.6)	71 (17.1)	13 (3.1)	11 (2.6)
United Kingdom	3,816	3,770		2,502 (65.6)	1,033 (27.1)	175 (4.6)	60 (1.6)
United States	2,614	2,397		1,225 (46.9)	982 (37.6)	65 (2.5)	125 (4.8)
Total	15,505	14,671		10,840 (69.9)	2,742 (17.7)	707 (4.6)	382 (2.5)

*All children were <18 years of age, except in the United Kingdom, where data were available only for children <17 years of age. No data were available from Australia. †Data from 1993–2002 only. ‡Data from 1998–2002 only. §Data from 1997–2002 only.

### Return to Native Country

High-risk malaria destinations reflect the migrant population in the source country. For France, >2 million travelers visited Africa; the overall rate of malaria acquisition for children was 22/10,000 arrivals in Africa and 110/10,000 arrivals in considerable-risk countries within Africa. However, travelers to specific countries had a huge comparative risk. Children from France who visited the Comoros islands (n = 10,460) had a malaria attack rate of 1,251/10,000. From France, the Comoros islands are a recognized destination for visiting friends and relatives; no other country evaluated reported any malaria cases for travelers to the Comoros. In comparison, large numbers of travelers from France, probably tourists, visit Kenya (n = 527,880), where the attack rate for the child travelers is 3.8/10,000 arrivals. The countries where children were most likely to acquire malaria were, in order of risk, Comoros (1,030 malaria cases/10,000 arrivals), Democratic Republic of Congo (778), Central African Republic (444), Guinea (308), Mali (203), Côte d’Ivoire (177), Congo (175), Nigeria (139), Bénin (134), Sierra Leone (127), Cameroon (109), Togo (102), and Ghana (100) and reflected the African nationality or origin of the immigrant communities in industrialized countries.

## Discussion

During 1992–2002, >17,000 cases of imported malaria in children were reported in 11 industrialized countries in which malaria is not endemic. Of all cases in children with known place of disease acquisition, >75% were acquired in Africa, mainly West Africa. *P. falciparum* was the dominant imported species; case-fatality ratio for all countries was <0.4%. Imported malaria in children is associated with travel, especially travel to visit friends and relatives, to high-risk malaria-endemic areas such as the Comoros islands and western and central African countries.

The strength of our study lies in the compilation of a large amount of data from national authorities, which enabled a global analysis. These data, coupled with data on arrivals in destination countries (*18*), enabled us to create a risk analysis for children traveling to malaria-endemic areas.

Limitations of our study include artifacts in malaria surveillance data and traveler statistics. Underreporting remains a problem in many countries (*19*), so our study could underestimate the true situation of imported malaria in children. Also, the quantity and quality of data received varied among countries and showed great heterogeneity despite efforts to standardize reporting in Europe (*3*).

Surveillance systems and malaria case definitions differ; some countries rely on laboratories or clinicians or both for source data (*19*). One country had data for children up to only 17 years of age, some countries had data for only part of the requested period, and some countries used extrapolated data.

Nationality and ethnicity posed logistical problems for data analysis. In the absence of data on “reason for travel,” we assumed that ethnicity represented the group who traveled to visit friends and relatives in their native country, which might not necessarily be true for countries such as the United States. The Figure shows the origin of the malaria patients rather than nationality, for which data are unavailable or unreliable. Information concerning the use of chemoprophylaxis is collected infrequently, if at all.

**Figure:**
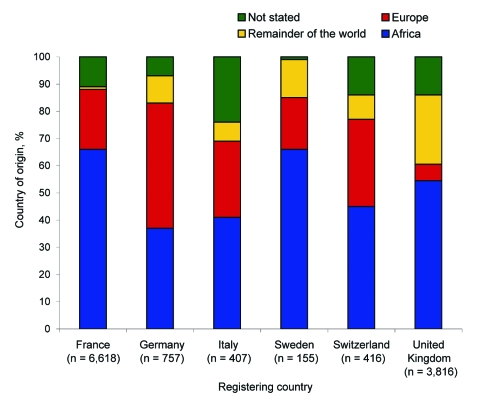
Country of origin of 12,214 children with imported malaria in 6 industrialized countries, 1992–2002.

In some countries, e.g., Germany, the Netherlands, and Australia, notification systems changed during the study period (*20*–*22*). Our collated cases have also been influenced by traveler’s choice of destination and use of preventive measures during travel, but other determinants included possible immunity or partial immunity of newly arrived immigrants to industrialized countries and number of travelers to malaria-endemic areas (*23*).

Statistics on traveler numbers, although imperfect, are the best available. To draw meaningful conclusions, we related numbers by destination country and source country to the corresponding visitation levels. Proxy data were used to estimate the percentage of young travelers to the various African destinations.

Data on arrivals need to be treated with caution because definitions, methods, and collection and compilation practices may differ from country to country. Many destination countries in Africa report arrivals by nationality and not by residency. With respect to malaria, method of reporting can imply that the number of visitors originating from the various source countries is higher than the actual number (nationals residing abroad might not be included) and that the number of malaria cases/10,000 children is overestimated. Furthermore, several destination countries have small focal areas where malaria transmission occurs, yet the denominator estimate included travel to the whole country, which may falsely lower the malaria risk estimate.

Our finding of a high rate of *P. falciparum* cases in France is consistent with findings of several studies about imported malaria in children in France (*13*,*14*,*24*,*25*). Castéla et al. describe malaria in France as essentially imported from Africa (*13*). Eloy et al. found that 90% of the 60 children with malaria at the Versailles Hospital between January 1997 and December 2001 were of African origin and that 84% had *P. falciparum* malaria (*26*).

In Italy, between 1989 and 1997, a steady increase in the number of cases among foreigners in all age groups has been reported, while cases among Italian nationals have remained stable (*27*). In 2000, foreign nationals represented almost 73% of total imported malaria cases in all age groups; of these, 93% were African (*28*). In our study, 41% of children with malaria registered in Italy were of African nationality. Place of acquisition of *P. falciparum* infection was Africa for >93% of children; >75% of cases were acquired in West Africa.

In the United Kingdom in the 1970s, a large proportion of imported malaria cases were attributable to *P. vivax* and associated with a large number of immigrants from India and Pakistan. Since the 1980s, however, the situation of imported malaria in the United Kingdom has changed (*29*,*30*); the overall ratio of cases caused by *P. falciparum* to those caused by *P. vivax* has increased from ≈37% in the mid-1980s to 55% in the mid-1990s (*15*). Of the 3,816 cases registered in the United Kingdom during 1992–2002, 65.6% were caused by *P. falciparum*, and 27.1% by *P. vivax.* The higher number corresponds with >50% of persons from Africa, compared with 25% from the Indian subcontinent.

In the United States, cases were usually imported from Central America and Asia by immigrants, as well as by US travelers. However, as in other countries where traditionally *P. vivax* has been imported, cases acquired in Central and South America and Asia decreased and cases acquired in Africa increased (*31*). Dorsey et al. found that most patients who imported malaria to the United States had become infected while in Central and South America (38% [35% and 3%, respectively]), followed by West and East Africa (31% [22% and 9%, respectively]), and Asia (29% [Indian subcontinent, 20%; Southeast Asia, 9%]) (*32*).

In general our findings support those reported in the literature and show that Africa plays a key role in importing malaria in children to industrialized countries where malaria is not endemic. In our study, of all imported malaria cases in children, >70% were acquired in Africa.

Imported malaria depends on the demographics of migrant populations and favored travel destinations of a country’s settled immigrant community, such as the Comorean community in France or the Nigerian community in the United Kingdom. At high risk for malaria are settled immigrants and their children who visit friends and relatives in their country of origin. Many migrants seem to mistakenly believe that they retain their partial immunity against malaria parasites, but immunity usually wanes rapidly (within 6 months) in the absence of exposure to *Plasmodium*-infected mosquitoes, although some immunologic memory for malaria may exist (*8*,*15*,*33*). In addition, parents of children born and raised in an industrialized country in which malaria is not endemic may mistakenly believe their children have partial immunity (*15*). Use of chemoprophylaxis is recommended for all children who travel to high-risk malaria-endemic areas (*34*). Several studies have indicated, however, that correct use of and adherence to chemoprophylaxis is low (*13*,*16*,*35*–*38*).

## Conclusions and Public Health Implications

Imported malaria in children is a complex problem that faces many challenges, including increasing global migrant and tourist travel; growing proportions of life-threatening falciparum malaria, combined with increasing resistance of malaria parasites to chemoprophylactic drugs; and lack of knowledge about and experience with imported malaria by physicians in industrialized countries where malaria is not endemic, which leads to delays in diagnosis and treatment of children with clinical malaria (*32*,*38*). The increasing proportions of *P. falciparum* cases are of relevance because *P. falciparum* malaria carries the greatest risk for life-threatening illness. Increasing *P. falciparum* resistance to antimalarial medication endangers the effectiveness of antimalarial chemoprophylaxis; therefore, standard recommendations for chemoprophylaxis need to be continually updated. Specific research on malaria among children who visit their native countries is warranted. These children are the most likely persons to acquire malaria yet the least likely to use adequate prevention strategies. Culturally sensitive approaches to malaria risk awareness and prevention are urgently needed for schools, the travel industry, and community groups. Local health authorities in communities with large ethnic minorities, particularly of African origin, need to recognize the problem of imported malaria. Some worthwhile community-based programs have been initiated. We conclude that malaria prevention for children should be a task of primary care providers and should be subsidized for low-income travelers to high-risk malaria-endemic areas.
